# Infrequent use of Public Access Defibrillation in spite of great benefit

**DOI:** 10.1186/1757-7241-23-S1-A7

**Published:** 2015-07-16

**Authors:** Marianne Agerskov, Anne M Nielsen, Mads W Jørgensen, Freddy K Lippert, Lars S Rasmussen

**Affiliations:** 1 Department of Anaesthesia, Centre of Head and Orthopaedics, Rigshospitalet, University of Copenhagen, Copenhagen, Denmark; 2 Emergency Medical Services, Copenhagen, Denmark; 3 Department of Cardiology, University of Copenhagen, Gentofte, Denmark

## Background

Despite wide dissemination of Public Access Defibrillation (PAD) and attempts to raise public awareness, use of Out of Hospital Cardiac Arrest (OHCA) is still limited. We aimed to study the use of PAD in Copenhagen. We primarily sought to determine the proportion of OHCA victims with an Automated External Defibrillator (AED) deployed before arrival of the Emergency Medical Services (EMS). In addition, we compared characteristics of OHCA victims according to use of AED.

## Methods

Between 2011 and 2013 we collected data on treated OHCAs in Copenhagen from the EMS, the Danish Cardiac Arrest Registry, and ECG downloads from deployed AEDs. Data on characteristics are reported as median values with Inter Quartile Range (IQR).

## Results

An AED was applied to an OHCA-victim prior to arrival of the EMS in 33 instances, corresponding to 3.7% of 903 AEDs registered in the voluntary network, http://www.hjertestarter.dk in Copenhagen. We identified 640 patients with treated OHCA in the EMS registry, of these 22 (3.4%, 95% CI [2.3-5.2]) had an AED applied, and 12 were defibrillated. Eleven of the 33 AED cases (33%) were not registered by the EMS. Six of these in fact had OHCA. Three had achieved Return Of Spontaneous Circulation (ROSC) upon arrival of the EMS and were not treated and 3 were declared dead. Four patients had no OHCA and one could not be identified. ROSC at hospital admission was significantly higher if an AED was applied (16/22 (73%) vs. 190/618 (31%), p < 0.0001). There was no significant difference in age ([61-77] vs. 68 [55-79] years, p = 0.64), response time (5 [4-7] vs. 5 [4-7] min, p = 0.41) or sex (male gender in 16 (73%) vs. 384 (62%), p = 0.38) according to AED deployment. An AED was applied in 4.0% of OHCAs in daytime vs. 2.7% during evening and night-time (p = 0.39).

## Conclusions

An AED was applied prior to EMS arrival in a minor proportion of OHCA and it could not be explained by differences in patient characteristics. However, a high survival rate to hospital admission of 73% in patients where an AED was used before EMS arrival indicates the potential and the need to overcome challenges in Public Access Defibrillation.

